# Study on a temperature-dependent selective soil treatment agent in regulating tobacco rhizosphere microbial community and controlling root and stem diseases

**DOI:** 10.3389/fmicb.2026.1717189

**Published:** 2026-03-23

**Authors:** Yuanhua Wu, Ming Fang, Xiaye Chen, Linghua Liu, Wenxin Wu, Lu Liu, Ting Liu, Shuo Xing, Weitao Wang, Guangliang Liu, Aiguo Chen, Zhaobao Wang

**Affiliations:** 1 Tobacco Research Institute of Chinese Academy of Agricultural Sciences, Qingdao, China; 2 Hunan Tobacco Company Chenzhou City Branch, Chenzhou, China; 3 Energy-rich Compounds Production by Photosynthetic Carbon Fixation Research Center, Shandong Provincial Key Laboratory of Microbial Resource Exploration and Innovative Utilization, College of Life Sciences, Qingdao Agricultural University, Qingdao, China; 4 China Tobacco Shandong Industrial Co., Ltd., Qingdao Cigarette Factory, Qingdao, China

**Keywords:** microbial community regulation, photosynthetic protection, root and stem diseases, selective soil treatment, tobacco

## Abstract

**Introduction:**

This study investigated the effects of a selective soil treatment agent on the microbial community structure of tobacco rhizosphere soil and the photosynthetic performance of tobacco plants at different temperatures (15 °C, 25 °C, 35 °C) through pot experiments.

**Methods:**

The control efficacy against black shank and root rot diseases was also evaluated.

**Results and discussion:**

The results indicated that 25 °C was the optimal temperature for the agent’s effectiveness. Under this condition, the agent significantly inhibited the enrichment of phytopathogenic fungi such as *Fusarium* spp. in the rhizosphere soil (the abundance of *Fusarium* in the T25SJ treatment decreased by 78.6% compared to the untreated group T25CK), while optimizing the microbial community structure, as evidenced by increased fungal *α*-diversity (Shannon index) and enhanced competitiveness of beneficial bacteria (e.g., *Pseudomonas* spp.). Notably, the bacterial community stability was maintained (no significant change in the Simpson index), confirming the agent’s selective regulatory effect rather than non-specific sterilization. Fluorescence imaging analysis revealed that the photosynthetic performance of tobacco plants treated with the agent at 25 °C was significantly higher than that of the untreated group, as showed by an increase in the maximum photochemical efficiency (Fv/Fm) (T25SJ vs. T25CK: 0.82 vs. 0.68), enhanced electron transport efficiency (ψ_Eo), and light energy conversion capacity (δ_Ro). In contrast, the untreated group exhibited severe damage to the photosynthetic system due to pathogen infection, leading to the inactivation of PSII reaction centers (increased ABS/RC and decreased RC/ABS values). At low (15 °C) and high (35 °C) temperatures, the agent still showed some inhibitory effects on certain pathogens (e.g., *Thielaviopsis* spp.), but the overall antibacterial efficiency and photosynthetic protection were significantly weaker than at 25 °C. Further analysis demonstrated that the agent alleviated oxidative stress caused by root damage and ensured the preferential allocation of light energy to photosynthesis by selectively suppressing dominant pathogens while preserving beneficial microbial populations. This study reveals that the antibacterial efficacy and photosynthetic protection of the soil treatment agent are significantly temperature-dependent, with 25 °C being the optimal application temperature. These findings provide a theoretical basis for the green control of soil-borne diseases in tobacco and the regulation of photosynthetic stress resistance, and offer a new paradigm for resolving the contradiction between “pathogen control” and “ecological protection” in soil-borne disease management.

## 1 Introduction

As an important cash crop, tobacco exerts a significant impact on China’s livelihood economy and military industry sectors (Hu, 2009). The growth status of tobacco at various developmental stages directly determines its economic value, among which temperature and diseases are two crucial limiting factors that persist throughout the entire growth cycle (Niu et al., 2025; Dudung Ahmad et al., 2024). First, in terms of temperature requirements, tobacco exhibits distinct stage-specific temperature thresholds, with the seedling stage being optimal at a temperature of 20–28 °C. Temperatures below 10 °C bring radicle growth to a halt, while temperatures above 35 °C leads to etiolated hypocotyl elongation and poor seedling vigor. The root elongation stage requires a soil temperature of 18–28 °C to promote the development of lateral roots and root hairs; excessively low temperatures will hinder the absorption of phosphorus and potassium, leading to reduced stress resistance of plants. The vigorous growth stage (from rosette to budding) is a critical window for the rapid accumulation of dry matter and aroma precursors; diurnal temperatures of 25–30 °C during the day and 16–20 °C at night, with a diurnal temperature range (DTR) of ≥8 °C, are most conducive to leaf thickening and achieving a leaf area index (LAI) of 3.2–3.5. Sustained temperatures above 35 °C, however, will induce premature ripening of tobacco, resulting in thinner leaves and poor oil content. During the maturation and curing stage, when the leaf temperature ranges from 32–42 °C, starch and proteins are rapidly degraded, forming a golden or orange color as well as a sweet and fresh fragrance; if the nighttime temperature is lower than 12 °C, the conversion of reducing sugars will be blocked, leading to dull color of cured leaves and a decline in commercial grade (Liu et al., 2024). It can be seen from the above that temperature directly determines the yield and grade of tobacco leaves by affecting photosynthetic intensity, substance translocation rate, and enzyme activity. Second, soil-borne diseases such as root rot (*Fusarium* spp.), bacterial wilt (*Ralstonia solanacearum*), and black shank (*Phytophthora nicotianae*) caused by fungi and bacteria, are characterized by strong concealment, rapid spread, and widespread complex infection (Fry, 2008; Larkin and Fravel, 1998; Panth et al., 2020). They often lead to large-scale death of tobacco plants and cause severe economic losses. As a major tobacco-producing country in the world, China has witnessed an intensification of damage caused by rhizosphere soil-borne diseases in some regions in recent years. Among these diseases, root rot is one of the main rhizosphere diseases, and its pathogenic flora is dominated by *Fusarium commune*, with the complex infection rate reaching 62.3% (Zhang and Ma, 2017). Field observations show that under high-temperature and high-humidity conditions from late March to mid-May, the complex infection of black shank and bacterial wilt can reduce tobacco field yield by 30–50% (Papadaki et al., 2017), which has become a crucial bottleneck restricting the improvement of tobacco leaf quality.

Moreover, the occurrence of diseases is highly coupled with temperature (Lafferty, 2009). Black shank (*Phytophthora nicotianae*) sees massive release of zoospores under the conditions of 20–24 °C and relative humidity > 90%, and outbreaks can occur within 7–10 days after transplanting in low-temperature and high-humidity environments (Bassani et al., 2020); bacterial wilt (*Ralstonia solanacearum*) has an optimal temperature range of 28–35 °C, sustained high temperatures accompanied by rainfall enable the bacteria to spread rapidly along vascular bundles, leading to whole-plant wilting within 3–5 days (An and Zhang, 2024); root black rot (*Thielaviopsis basicola*) is most active when soil temperature is 25–30 °C and soil is alkaline. Temperature not only determines the infection window of pathogens but also amplifies disease risks by altering the physiological state of the host and the rhizosphere microecology (Qurrat Ul Ain et al., 2024). Additionally, temperature and diseases exert a significant interactive effect, and on one hand, temperature determines the length of the incubation period. For example, the incubation period of black shank disease is only 3–4 days at 22 °C, but extends to 8–10 days when the temperature drops below 18 °C, which gains extra time for chemical control. On the other hand, continuous high or low-temperature stress inhibits the activity of key enzymes (PAL, POD) in the phenylpropanoid metabolism pathway, hindering the synthesis of lignin and pathogenesis-related proteins and thereby making it easier for *Ralstonia solanacearum* to penetrate the root cortex (Ge, 1996; Li et al., 2019). Besides the temperature factor, the occurrence and development of soil-borne diseases are also regulated by other multiple ecological factors. In terms of climatic factors, the optimal infection temperature for pathogens such as *Phytophthora* spp. and *Ralstonia solanacearum* is concentrated in the range of 20–30 °C, and the spread of their zoospores shows a significant positive correlation with rainfall (Sbeiti et al., 2023; Li et al., 2017); in terms of soil microecology, the imbalance of microbial communities in continuously cropped tobacco fields leads to the accumulation of pathogen abundance, and the relative abundance of *Fusarium* spp. in typical diseased fields can reach 8.6 times that of healthy soil (Yang et al., 2025). Additionally, soil-borne diseases also exert a severe impact on the performance of tobacco plants. Pathogens infect the root system and stem base of tobacco plants, destroying vascular bundle tissues and impairing water absorption; this leads to leaf water deficiency, a decrease in chlorophyll content, and damage to the photosystem. Under natural disease occurrence conditions, as the disease severity increases, various photosynthetic indices of tobacco plants are inhibited (Wang et al., 2022). Therefore, how to inhibit rhizosphere soil-borne diseases of tobacco is one of the key factors for the sustainable development of tobacco leaf production.

Although traditional chemical control can suppress plant diseases in the short term, it is associated with critical issues, including enhanced pathogen resistance, soil residue accumulation, and indiscriminate disinfection (Aref, 2020). In fact, empirical data have demonstrated that the disease control efficiency drops to below 30% after the continuous application of the same fungicide over three growing seasons (García-Enciso et al., 2017). Moreover, sole reliance on chemical-based approaches is often poorly sustainable, as such methods not only pose potential risks to the environment and human health but also may exert adverse effects on host plants and even lead to increased carbon emissions (Castello et al., 2024, 2025). What is more severe is that broad-spectrum soil fumigants (e.g., dazomet) cause “soil ecological vacuum” by non-specifically killing both pathogenic and beneficial microbes, disrupting soil fertility and ecological resilience (Gao et al., 2022). As an environment-friendly prevention and control method, biological control exhibits unique advantages in improving system disease resistance by introducing antagonistic microbial communities to reconstruct the soil microbial interaction network (Chen et al., 2020). However, in field applications, it has been found that the colonization efficiency of biocontrol agents is insufficient (fewer than 15% of strains can survive stably for 28 days) and the bacteriostatic effect fluctuates significantly (the coefficient of variation of control efficiency reaches 35.6%) (del Lorena Rosario et al., 2023). The underlying reasons are as follows, competitive exclusion by indigenous microorganisms, spatiotemporal heterogeneity of soil physical and chemical properties, and formation of pathogen biofilms (Delitte et al., 2024).

To address the core contradiction between “pathogen control” and “ecological protection” in soil-borne disease management, this study proposes a synergistic prevention and control strategy of “selective suppression, ecological reconstruction”. We employed a selective soil treatment agent independently developed by the Tobacco Research Institute of the Chinese Academy of Agricultural Sciences, which targets metabolically active pathogens while preserving beneficial microbial populations and soil microbial community stability. Unlike traditional broad-spectrum disinfectants, this agent’s design principle is based on differential niche attack. It prioritizes targeting pathogenic microbes in active infection states (e.g., germinating spores, zoospores, or active hyphae) with relatively weak antioxidant capacity, while having limited impact on dormant or antioxidant-rich beneficial microbes. On this basis, establish a microecological environment suitable for the colonization of biocontrol microbial communities. This study conducted a pot experiment to investigate the effects of soil treatment agents on the microbial community structure in tobacco root soil and the photosynthesis of tobacco plants under different temperature conditions. We compared the changes in soil microbial community structure before and after treatment to analyze the inhibitory efficacy of this soil treatment agent against key pathogenic genera including *Fusarium* spp. and *Phytophthora* spp. The preservation of beneficial microbes and community stability were also analyzed. Furthermore, the photosynthetic performance between the treated and untreated groups was compared to analyze the impact of this treatment product on plant photosynthesis. Findings from this study provide a theoretical basis for overcoming the application barriers of biological control agents and establishing a green prevention and control technology system.

## 2 Materials and methods

### 2.1 Experimental materials

The tested soil was collected from a tobacco field with diseased plants at the mature stage in Renyi Town, Guiyang County, Chenzhou City, Hunan Province, China (112°13′26″E to 112°55′46″E, 25°37′15″N to 26°13′30″N), in May 2024. During soil collection, the diseased plant was taken as the central point, and soil samples with a width of 15 cm on one side and a depth of 30 cm were collected, mixed uniformly, and stored under freezing conditions. The pot experiment was conducted in the artificial climate chamber of the Jimo Experimental Base, Tobacco Research Institute of the Chinese Academy of Agricultural Sciences (CAAS). The tobacco variety used in the experiment was Yunyan 87.

The soil treatment agent used in this study is an independently developed product by the Tobacco Research Institute of the Chinese Academy of Agricultural Sciences (CAAS). Its reaction mechanism involves the release of atomic oxygen, hydroxyl free radicals, and other reactive species by the product, which destroy the internal structures of soil-dwelling bacteria, fungi, viruses, and insect eggs.

### 2.2 Experimental design and procedures

#### 2.2.1 Experimental design

Three temperature gradient treatments were set up, namely T15: 15 °C (temperature control ±1 °C), T25: 25 °C (temperature control ±1 °C), and T35: 35 °C (temperature control ±1 °C). Under each temperature gradient, two treatment groups were established, the agent treatment group (SJ) and the untreated control group (TCK). Additionally, the original soil sample (before any cultivation) was used as the blank control (CK).

#### 2.2.2 Experimental procedures

Soil sample pretreatment was conducted as follows. Impurities such as stones and plant residues were removed from the collected soil samples. After thorough mixing, a portion of the soil sample was collected using the multi-point sampling method as the original soil sample (CK). The original soil sample was quickly frozen in liquid nitrogen and then stored in an ultra-low temperature refrigerator at −80 °C for later use.

Soil treatment was conducted as follows. The remaining soil sample was evenly divided into two parts. One part was treated with the treatment product (SJ), and the other part was treated with an equal volume of clear water as the untreated control group (TCK).

#### 2.2.3 Pot setup

The treated soil was filled into flower pots with an upper diameter of 16 cm, a lower diameter of 13 cm, and a height of 17.5 cm. One robust tobacco seedling with uniform growth was transplanted into each pot, and each treatment was set with 6 replicates.

#### 2.2.4 Cultivation conditions

The potted plants were placed in an artificial climate incubator with the preset temperatures. A water tray was placed under each pot, and the water level was maintained at approximately 1 cm to ensure that the soil moisture was kept at 80–90% of the field water capacity. The incubator was set with a light cycle of 12 h light/12 h dark to simulate the natural day-night alternation. The pot trials were conducted as three independent replicates.

### 2.3 Index determination and methods

#### 2.3.1 Observation and recording

In accordance with GB/T 23222—2008 *Grading and Investigation Methods for Tobacco Diseases and Pests*, the disease occurrence of different treatments was recorded through continuous observation. In the initial stage of the disease, tobacco plants were observed and recorded on a weekly basis. When any tobacco plant developed the disease to Grade 3, the observation and recording were adjusted to a daily frequency until any tobacco plant reached Grade 4 of disease severity.

#### 2.3.2 Soil DNA sequencing analysis

When tobacco plants in the experimental treatments developed to disease grade 4, rhizosphere soil samples were collected from all treated tobacco plants (n ≥ 3), treated with liquid nitrogen, and then stored in a − 80 °C refrigerator. Total soil DNA was extracted, and PCR amplification was performed targeting the bacterial 16S rRNA gene and fungal ITS region. High-throughput sequencing was conducted using the Illumina platform. Data analysis included sequence preprocessing, OTU/ASV clustering, and diversity analysis [OTU (Operational Taxonomic Unit); ASV (Amplicon Sequence Variants)] to study the structure and diversity of the microbial community.

Total soil DNA was extracted using the CTAB method. The V3-V4 hypervariable region of the bacterial 16S rRNA gene was amplified with primers 338F (5’-CTCCTACGGGAGGCAGCAG-3′)/806R (5’-GGACTACHVGGGTWTCTAAT-3′), and the fungal ITS1 region was amplified with primers ITS5-1737F (5’-GGAAGTAAAAGTCGTAACAAGG-3′)/ITS2-2043R (5’-GCTGCGTTCTTCATCGATGC-3′). After the PCR products were verified as qualified by agarose gel electrophoresis, a paired-end (PE) 250/PE 300 sequencing library was constructed, and high-throughput sequencing was performed on the Illumina NovaSeq 6000 platform.

After the raw data were quality-controlled and filtered using Trimmomatic, the DADA2 algorithm was used for denoising and generating ASVs (Amplicon Sequence Variants). Species annotation was conducted based on the Silva 138 (bacteria) and UNITE 8.3 (fungi) databases. For *α*-diversity analysis, the Shannon, Chao1, and Simpson indices were calculated. For *β*-diversity analysis, PCoA (Principal Coordinates Analysis) and PERMANOVA (Permutational Multivariate Analysis of Variance) tests were performed based on the Bray-Curtis distance matrix (using the QIIME2 platform). LEfSe (Linear Discriminant Analysis Effect Size) analysis (LDA > 3.5) was used to screen for differential characteristic microorganisms between groups, and Spearman correlation analysis was used to explore the correlation between microbial community structure and the disease severity of tobacco plants. All statistical analyses were completed in the R 4.2.1 environment, and data visualization was implemented using the ggplot2 package.

The data used in this work are openly available in the NCBI Sequence Read Archive (SRA) at: https://www.ncbi.nlm.nih.gov/bioproject/1337005 and the reference number is PRJNA1337005.

#### 2.3.3 Photosynthetic efficiency and performance analysis

Tobacco seedlings from each treatment group (*n* ≥ 5) were placed in a constant temperature environment of 25 °C for 30 min of dark adaptation, and then detected using a multi-channel chlorophyll fluorescence imaging system (IMAGING-PAM M series, Walz, Germany). The measurement parameters were set as follows, measuring light intensity of 10 μmol·m^−2^·s^−1^, saturated pulse light intensity of 3,000 μmol·m^−2^·s^−1^ (pulse width 0.8 s), and actinic light intensity of 200 μmol·m^−2^·s^−1^.

The minimum fluorescence (F₀), maximum fluorescence (F_m_), and dynamic fluorescence signals were captured using the supporting software (ImagingWin v2.56) to obtain the OJIP fast chlorophyll fluorescence induction curve. Based on the standardized JIP-test model (Strasser et al., 2000), the following key parameters were calculated:


$$\begin{array}{l} \text{Maximum photochemical efficiency of}\\ \text{photosystem}\;\mathrm{II}\;(\text{PSII}):\mathrm{Fv}/\mathrm{Fm}=\frac{\left(\mathrm{Fm}-\mathrm{Fo}\right)}{\mathrm{Fm}} \end{array}$$ (1)

$$\begin{array}{l} \text{Electron transfer efficiency},\psi \_\mathrm{Eo}\;\\ (\mathrm{ETo}/\mathrm{TRo}):\mathrm{\psi Eo}=\frac{\mathrm{FJ}-\mathrm{Fo}}{\mathrm{Fm}-\mathrm{Fo}} \end{array}$$ (2)

$$\begin{array}{l} \text{Efficiency of electron acceptors}\;\mathrm{at}\;\text{the}\;\mathrm{end}\;\text{of}\;\\ \mathrm{PSI},\delta \_\mathrm{Ro}\;(\mathrm{Reo}/\mathrm{ETo}):\delta_{\mathrm{Ro}}=\frac{\mathrm{FI}-\mathrm{FJ}}{\mathrm{Fm}-\mathrm{FJ}} \end{array}$$ (3)

$$\begin{array}{l} \text{Light energy capture efficiency},\varphi \_\mathrm{Po}\;\\ (\mathrm{TRo}/\mathrm{ABS}):\mathrm{\varphi Po}=\frac{\left(\mathrm{Fm}-\mathrm{Fo}\right)}{\mathrm{Fm}} \end{array}$$ (4)

$$\begin{array}{l} \text{Performance Index}\;(\mathrm{PI}\_\mathrm{ABS}),a\;\text{comprehensive}\;\\ \text{performance index reflecting the overall activity of}\\ \text{PSII}:\mathrm{PI}\_\mathrm{ABS}=\frac{\mathrm{RC}}{\mathrm{ABS}}\times \frac{\mathrm{\varphi Po}}{1-\mathrm{\varphi Po}}\times \frac{\mathrm{\psi Eo}}{1-\mathrm{\psi Eo}} \end{array}$$ (5)

#### 2.3.4 Statistical analyses

One-way ANOVA combined with Tukey’s HSD multiple comparison test (at a significance level of *p* < 0.05) was employed to analyze the differences in photosynthetic performance indices among different treatment groups. Prior to data analysis, the Shapiro–Wilk normality test and Levene’s test for homogeneity of variances were conducted to confirm that the data met the applicable requirements of ANOVA. The significance of differences revealed by the Tukey’s HSD test was represented using the letter labeling method, where the same letter indicates no significant difference among groups and different letters indicate a significant difference among groups. The number of biological replicates for all treatment groups was n = 6, and all statistical analyses were performed using SAS 9.1 software.

## 3 Results and analysis

### 3.1 Effect of different treatments on the composition of microbial community in tobacco rhizosphere soil

**Figure 1:**
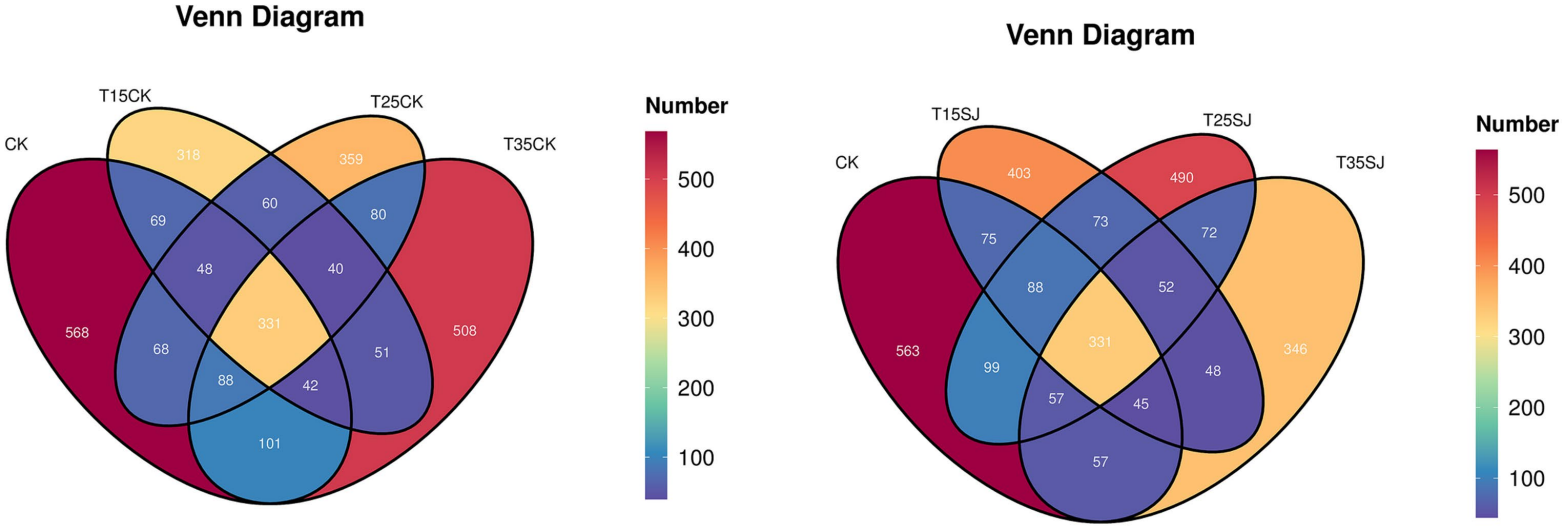
Venn diagram of fungal community.

**Figure 2:**
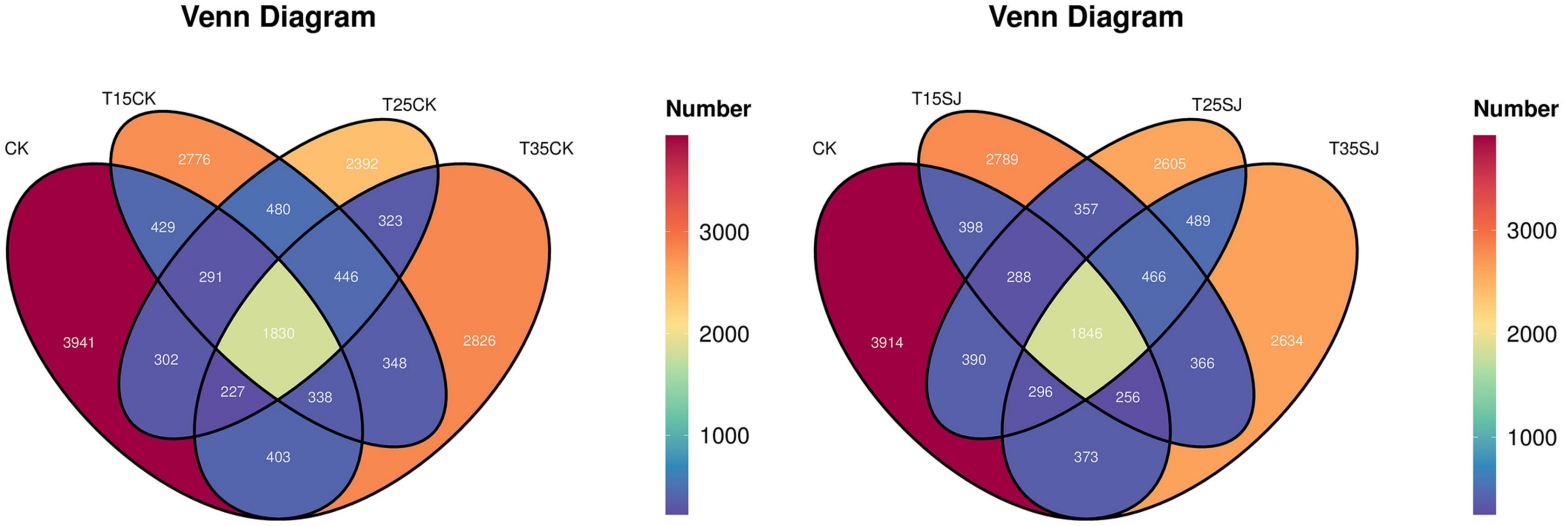
Venn diagram of bacterial community.

From the Venn diagram of the fungal community (Figure 1), it can be observed that among the original soil sample (CK) and the untreated cultivated soil samples (TCK series), the number of fungal ASVs (Amplicon Sequence Variants) was 568 for CK, 508 for T35CK, 359 for T25CK, and 318 for T15CK, with a shared number of 331 ASVs across all groups. Regarding the unique number of fungal ASVs between the original soil sample (CK) and the treatment soil samples (SJ series), the values were 563 for CK, 346 for T35SJ, 490 for T25SJ, and 403 for T15SJ, with a shared number of 331 ASVs. These results indicate that the application of the soil agent exerts a certain impact on the structure of soil fungal microorganisms. As the Venn diagram of the bacterial community (Figure 2), the comparative analysis revealed that the number of bacterial ASVs in the original soil sample (CK) was higher than that in all treatment soil samples (TSJ series), with the order being CK (3914) > T15SJ (2789) > T35SJ (2634) > T25SJ (2605). The analysis also indicated that the number of bacterial ASVs in CK exceeded that in all untreated cultivated soil samples (TCK series), with the order being CK (3941) > T35CK (2826) > T15CK (2776) > T25CK (2392). These findings suggest that under the 25 °C cultivation condition, the application of the soil agent has the most significant effect on altering the number of bacterial ASVs, while the effect at 35 °C is less pronounced than that at 15 °C.

### 3.2 α-diversity analysis of rhizosphere soil microorganisms under different treatments

**Figure 3:**
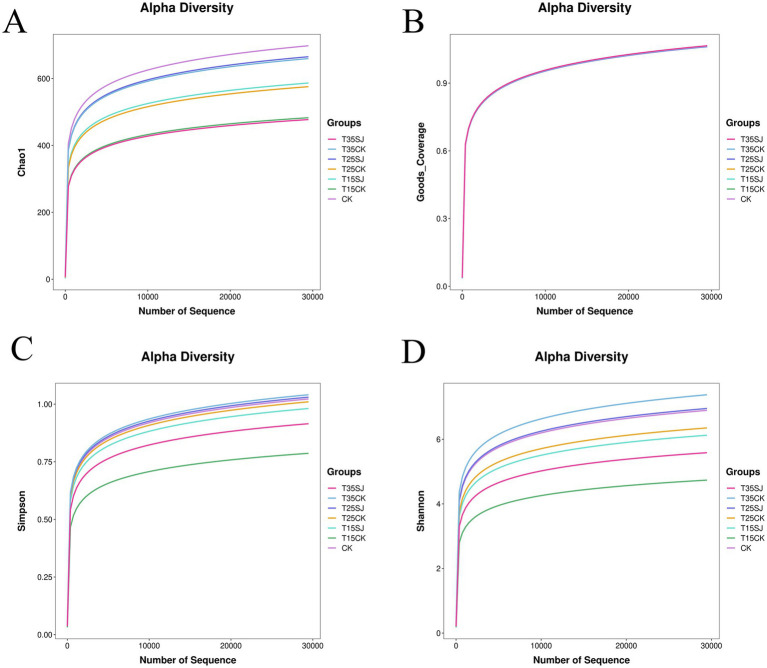
Rarefaction curve analysis of *α*-diversity of soil fungal microorganisms. **(A)** Microbial community richness index; **(B)** sample coverage index; **(C)** soil microbial species diversity; **(D)** Shannon diversity index.

To reveal the response of soil microorganisms to different treatment groups, α-diversity analysis was used to evaluate the richness and diversity of soil fungal microorganisms among different treatment groups (Table 1). The results showed that the coverage index of all samples was close to 1 (Figure 3B), indicating sufficient sample coverage. The ranking of fungal α-diversity (comprehensive index) was as follows, CK > T25SJ > T35CK > T15SJ > T25CK > T15CK > T35SJ. This result suggests that the soil agent can affect the richness of the fungal community. For the Shannon index (a key index reflecting microbial diversity, Figure 3D), the ranking was T35CK > T25SJ > CK > T25CK > T15SJ > T35SJ > T15CK. However, no significant difference was demonstrated, indicating the treatments did not impair the overall diversity and structural stability of the soil bacterial community.

**Table 1:** Analysis of α-diversity of soil fungal microorganisms.

Treatment	Shannon^(ns)^	Simpson^(ns)^	Chao1^(ns)^	Coverage^(ns)^
CK	6.45 ± 0.17	0.95 ± 0.01	668.53 ± 59.55	0.9982 ± 0.0006
T15CK	4.42 ± 1.42	0.73 ± 0.21	466.54 ± 92.43	0.9983 ± 0.0003
T15SJ	5.73 ± 0.21	0.91 ± 0.02	571.25 ± 7.04	0.9981 ± 0.0005
T25CK	5.94 ± 0.17	0.94 ± 0.01	550.09 ± 13.10	0.9986 ± 0.0005
T25SJ	6.51 ± 0.18	0.96 ± 0.01	644.37 ± 12.17	0.9983 ± 0.0006
T35CK	6.91 ± 0.05	0.97 ± 0.01	626.56 ± 55.61	0.9993 ± 0.0005
T35SJ	5.23 ± 1.53	0.85 ± 0.12	461.65 ± 150.90	0.9989 ± 0.0003

ns, no significant difference among treatments (*p* > 0.05).

**Figure 4:**
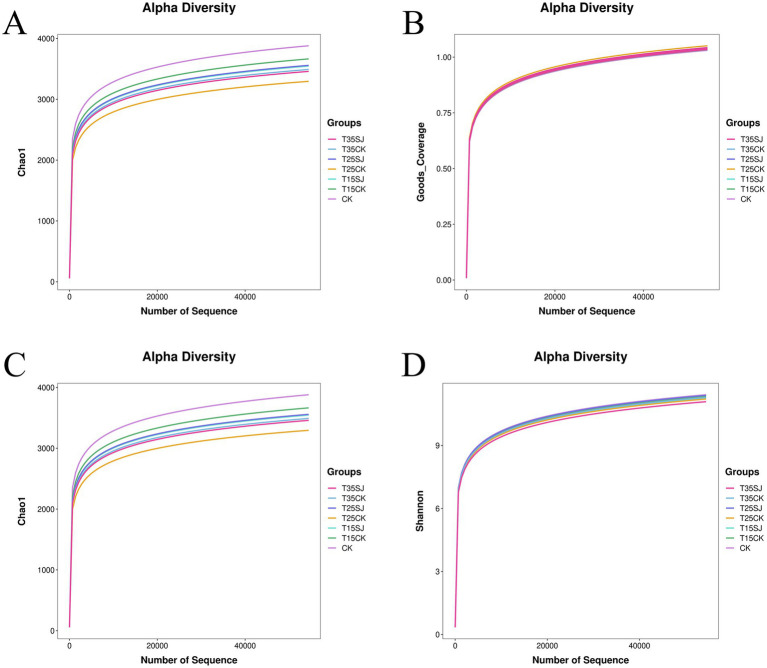
Rarefaction curve analysis of α-diversity of soil bacterial microorganisms. **(A)** Microbial community richness index; **(B)** sample coverage index; **(C)** soil microbial species diversity; **(D)** Shannon diversity index.

As shown in Table 2 and Figure 4, the rarefaction curves of all data related to microbial community structure diversity tended to flatten, which confirms that the amount of detection data has reached saturation (i.e., further increasing the sequencing depth will not significantly increase the number of observed species). The Simpson index (an index reflecting community evenness) remained stable across all treatments. For the Chao1 index (a key index representing microbial richness), the values showed the following trend, T15SJ < T15CK, T25SJ < T25CK, and T35SJ < T35CK. Additionally, the bacterial microbial richness in the treatment soil was lower than that in the blank control (CK) across all temperature gradients. These results indicate that although the number of bacterial species decreased after treatment, the distribution of the remaining species was more uniform. Collectively, the results demonstrate that the soil agent exhibits a significant selective inhibitory effect on dominant pathogenic bacteria.

**Table 2:** Analysis of α-diversity of soil bacterial microorganisms.

Treatment	Shannon^(ns)^	Simpson^(ns)^	Chao1^(ns)^	Coverage^(ns)^
CK	10.77 ± 0.23	0.9986 ± 0.0002	3669.85 ± 275.79	0.9983 ± 0.0002
T15CK	10.69 ± 0.13	0.9984 ± 0.0003	3479.08 ± 332.35	0.9983 ± 0.0004
T15SJ	10.62 ± 0.06	0.9984 ± 0.0001	3359.08 ± 76.61	0.9988 ± 0.0005
T25CK	10.56 ± 0.43	0.9982 ± 0.0010	3116.65 ± 654.73	0.9994 ± 0.0005
T25SJ	10.73 ± 0.08	0.9987 ± 0.0001	3367.28 ± 202.06	0.9991 ± 0.0007
T35CK	10.64 ± 0.06	0.9986 ± 0.0001	3303.88 ± 183.02	0.9994 ± 0.0001
T35SJ	10.44 ± 0.54	0.9962 ± 0.0033	3277.46 ± 388.21	0.9986 ± 0.0004

ns, no significant difference among treatments (*p* > 0.05).

### 3.3 β-diversity analysis of rhizosphere soil microorganisms under different treatments

**Figure 5:**
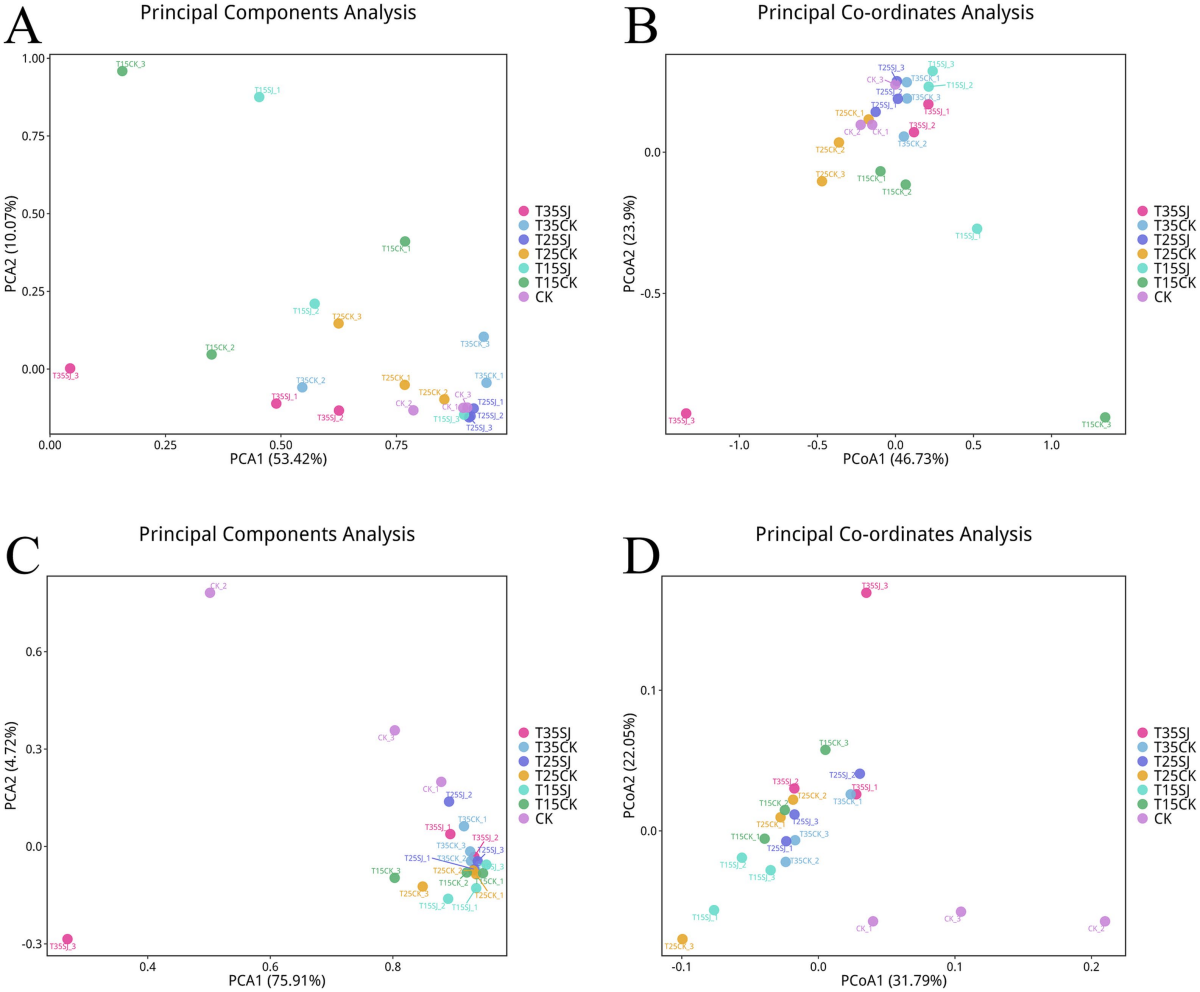
Analysis of *β*-diversity of soil microbial communities. **(A)** Principal component analysis (PCA) of fungal communities; **(B)** weighted-Unifrac principal coordinates analysis (PCoA) of fungal communities; **(C)** PCA of bacterial communities; **(D)** PCoA of bacterial communities.

Principal Component Analysis (PCA) and Principal Coordinates Analysis (PCoA) were performed to assess the β-diversity of soil fungal and bacterial communities (reflecting differences in community composition among groups). For the fungal community, the first principal component (PC1) and the second principal component (PC2) accounted for 53.42 and 10.07% of the total variation, respectively (see Figure 5A). In the PCoA, the first coordinate (PCoA1) and the second coordinate (PCoA2) explained 46.72 and 23.90% of the community variation, respectively (Figure 5B). Notably, the fungal community compositions of the T35SJ, T15CK, T15SJ, and T25CK groups showed significant differences from those of the other treatment groups. These results were similar with the findings from the β-diversity of the bacterial community (Figures 5C,D). Collectively, these results indicate that the effect of applying the soil agent under the temperature conditions of 35 °C (T35SJ) and 15 °C (T15SJ) is superior to that under 25 °C (T25SJ) in terms of altering the fungal community structure.

### 3.4 Species composition and difference analysis of rhizosphere soil microorganisms under different treatments

**Figure 6:**
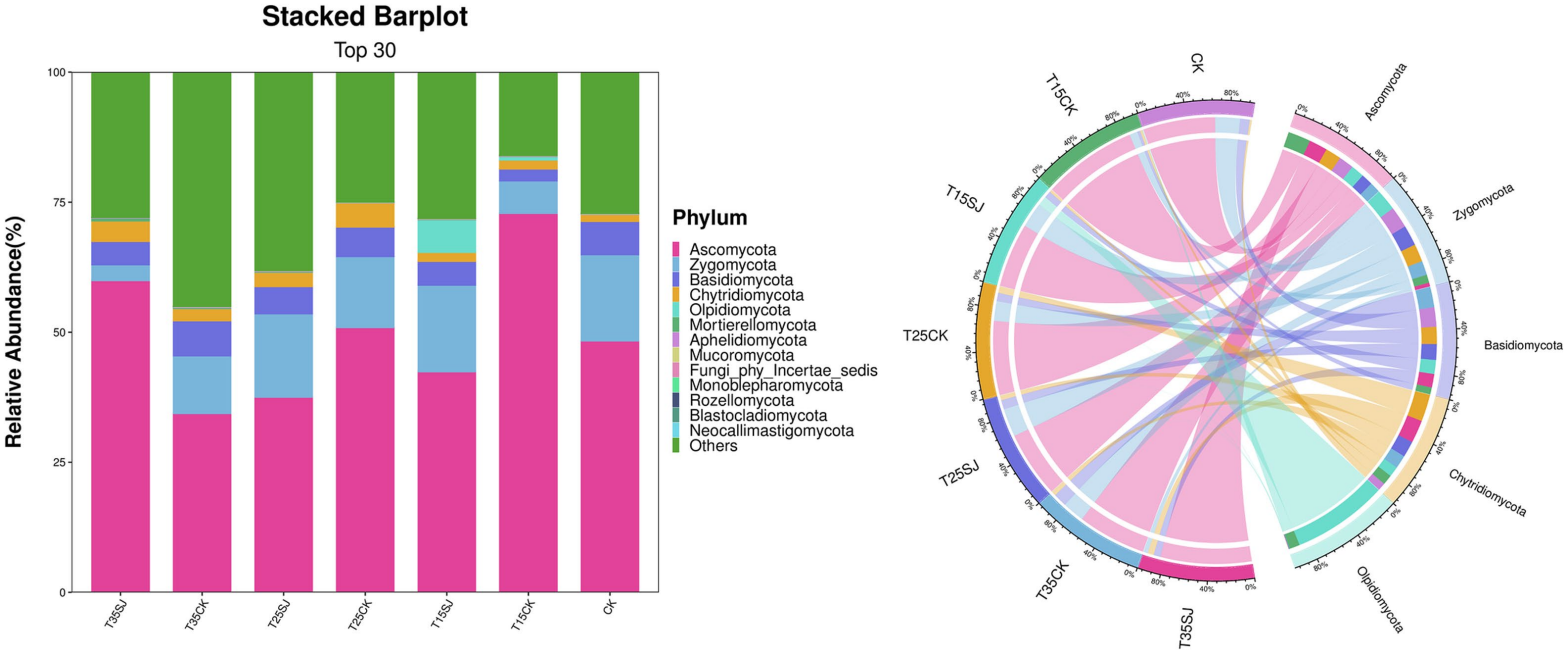
Composition of soil fungal microorganisms at the phylum level in different samples.

**Figure 7:**
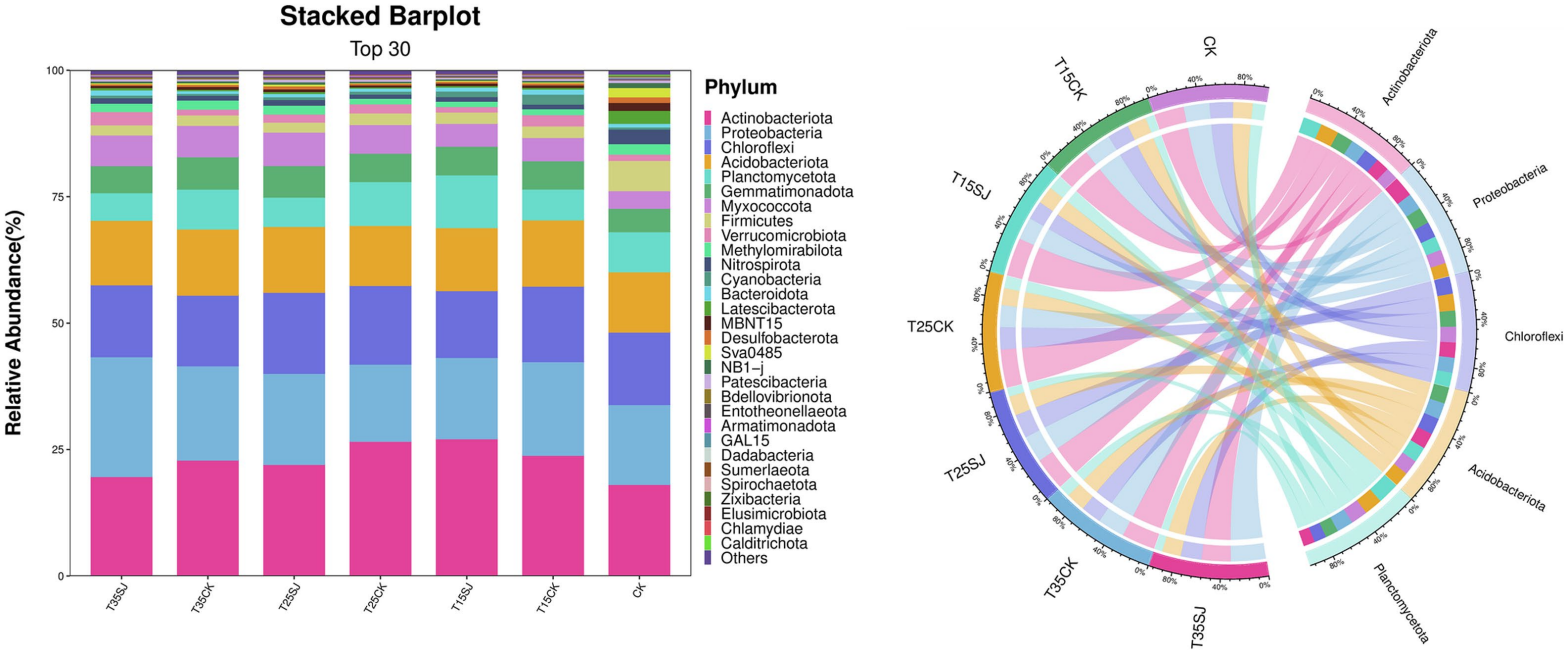
Composition of soil bacterial microorganisms at the phylum level in different samples.

At the phylum level, the top-ranked fungal phyla in the soil included *Ascomycota, Zygomycota, Basidiomycota, Chytridiomycota,* and *Olpidiomycota*, which accounted for 60–80% of the total soil fungi in each sample (Figure 6). Among them, *Ascomycota* was the first dominant phylum, accounting for approximately 30–55% of the total. The T15CK group had the highest abundance, accounting for 72.87% of the total, followed by the T35SJ group, accounting for 59.94% of the total. The abundances of the other treatment groups were T25CK (50.93%), CK (48.34%), T15SJ (42.42%), T25SJ (37.54%), and T35CK (34.39%), respectively. There was a significant difference in this phylum between the T15CK and T15SJ treatment groups. *Zygomycota* was the second dominant phylum, accounting for approximately 3–17% of the total. The abundances of each group were T35SJ (3.03%), T35CK (11.08%), T25SJ (16.02%), T25CK (13.64%), T15SJ (16.64%), T15CK (6.25%), and CK (16.58%), respectively. Among them, there were significant differences in this phylum between T35SJ and CK, as well as between T15CK and CK. *Basidiomycota* was the third dominant phylum; the proportions of T35SJ, T35CK, T25SJ, T25CK, T15SJ, T15CK, and CK treatments were 4.5, 6.76, 5.23, 5.67, 4.58, 2.28, and 6.41%, respectively. There was no statistical significance in the difference between groups, indicating that this phylum remained relatively stable in the soil after treatment. Different treatments exerted a significant impact on the bacterial community in tobacco rhizosphere soil relative to the CK group. The relative abundance of the top 5 bacterial phyla detected in tobacco rhizosphere soil accounted for approximately 70% of the total bacterial community (Figure 7), including *Actinobacteriota, Proteobacteria, Chloroflexi, Acidobacteriota,* and *Planctomycetota.* The proportions of these five dominant bacterial phyla were similar among different treatment groups, and there was no statistical difference between groups.

### 3.5 Analysis of pathogenic flora causing root rot and black shank in rhizosphere soil under different treatments

**Figure 8:**
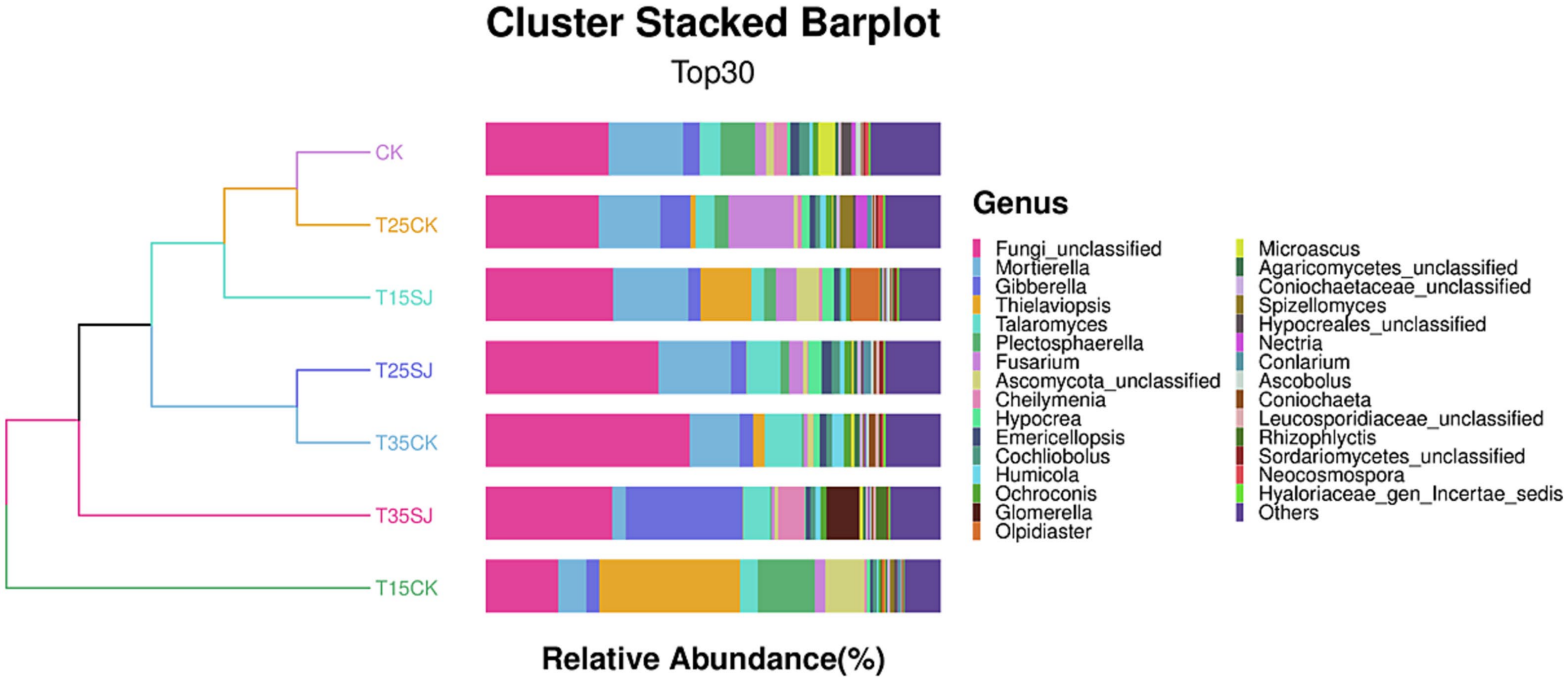
Composition of soil fungal microorganisms at the genus level in different samples.

**Figure 9:**
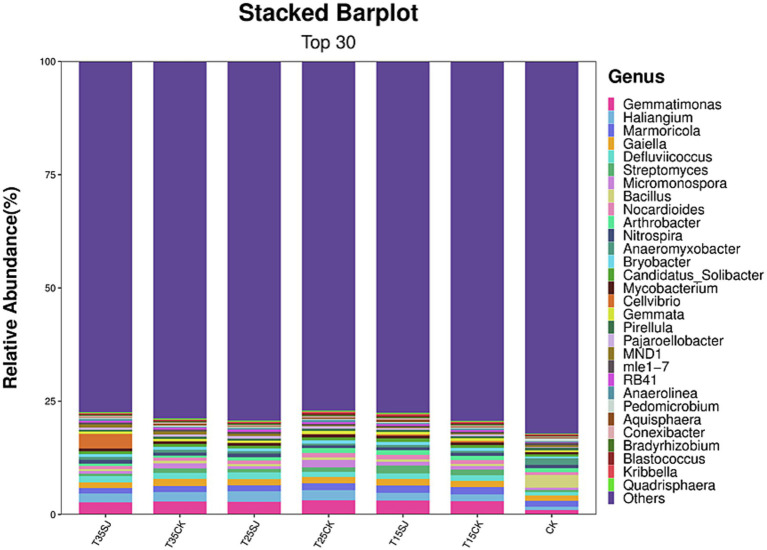
Composition of soil bacterial microorganisms at the genus level in different samples.

Analysis of the top 30 fungal genera (ranked by relative abundance) in different soil samples revealed significant differences in fungal composition among different treatment groups. As shown in Figure 8, *Fusarium*, the pathogenic genus responsible for root rot, was significantly enriched at 25 °C, with a relative abundance of 14.42% in the T25CK group. In contrast, its abundance in the T25SJ group (3.09%) was much lower, indicating that the application of the soil agent could significantly reduce the enrichment of *Fusarium* at 25 °C. Notably, the abundance of *Fusarium* in the T15SJ group (4.52%) was slightly higher than that in the T15CK group (2.34%), suggesting that the agent had a poor inhibitory effect on *Fusarium* at 15 °C. For the 35 °C treatment, the abundance of *Fusarium* in T35SJ (0.71%) was lower than that in T35CK (0.89%), indicating a certain inhibitory effect under high-temperature conditions, though the effect was not significant. *Thielaviopsis* (the pathogenic genus easily causing tobacco black root rot) was significantly enriched under the 15 °C temperature condition. Comparison between the T15SJ and T15CK groups showed that the application of the soil agent could reduce the enrichment of this genus. The pathogenic microorganisms causing tobacco black shank mainly include bacteria and fungi. The main fungal pathogenic genera are *Phytophthora nicotianae*, *Fusarium*, and *Rhizopus*, while the main bacterial genus is *Streptomyces*. As shown in Figure 9, *Phytophthora nicotianae* (tobacco black shank pathogen) did not appear among the top 30 fungal genera in this experiment. The abundances of *Fusarium* in each treatment group were CK (2.41%), T15CK (2.34%), T15SJ (4.52%), T25CK (14.42%), T25SJ (3.09%), T35CK (0.89%), and T35SJ (0.71%). Under the 25 °C condition, the addition of soil agent significantly reduced the enrichment of *Fusarium* in the soil; the abundance of *Fusarium* in T35SJ was slightly lower than that in T35CK, while the inhibitory effect was not obvious at 15 °C. There was no significant difference in the abundance of *Rhizopus* among different treatment groups. The abundances of *Streptomyces* in each treatment group were CK (0.46%), T15CK (1.24%), T15SJ (1.72%), T25CK (0.99%), T25SJ (0.75%), T35CK (0.99%), and T35SJ (0.52%). Comprehensive analysis of the pathogenic genera causing black shank showed that the application of soil agent had the most obvious regulatory effect at 25 °C. In conclusion, the application of soil agent exhibited the most significant inhibitory effect on tobacco root rot and black shank at 25 °C, while it exerted a certain inhibitory effect on some pathogenic genera under low-temperature (15 °C) and high-temperature (35 °C) conditions.

### 3.6 Analysis of damage degree of tobacco photosynthetic system under disease stress

At the initial stage of tobacco plants under disease stress, the plants activate a protective mechanism by slightly decreasing QY_max (maximum quantum yield) and increasing Dlo/RC (dissipated energy flux per reaction center). As the disease continues to infect, Phi_ET2o (electron transport efficiency) and Phi_Po (maximum photochemical efficiency of PSII) (Equation 1) decrease significantly, while ABS/RC (absorbed energy flux per reaction center) increases, leading to the inactivation of PSII (Photosystem II) reaction centers. Until the late stage of infection, Fv/Fo (ratio of variable fluorescence to minimum fluorescence) decreases sharply, and the RC/ABS (reaction centers per absorbed energy) value drops to an extremely low level, resulting in the collapse of the photosynthetic system. As shown in Table 3, the values of QY_max, Phi-Po, Phi-ET1o, Fv/Fo, and Mo in the T25CK group were significantly lower than those in the T25SJ group, and there were statistically significant differences between the two groups.​ The values of Psi-RE1o (efficiency of electron transfer to PSI end acceptors), ABS/RC, and Dlo/RC in the T25CK group were significantly higher than those in the T25SJ group, and the differences were also statistically significant. The PC/ABS value in the T25CK group was lower than that in the T25SJ group, but the difference was not statistically significant. In contrast, under the 15 °C temperature condition, there were no obvious differences in the above indicators among different treatment groups. These results indicate that the infection of pathogenic bacteria has a relatively slight impact on tobacco plants under low-temperature conditions.

**Table 3:** Analysis of photosynthetic performance of tobacco plants under different treatments.

Treatment	QY_max	Phi_Po	Phi_ET2o	Psi_RE1o^(ns)^	ABS/RC^(ns)^	RC/ABS^(ns)^	TRo/RC^(ns)^	DIo/RC^(ns)^	Mo^(ns)^
T15CK	0.80 ± 0.01a	0.80 ± 0.01a	0.59 ± 0.71a	0.29 ± 0.01	1.64 ± 0.06	0.62 ± 0.16	1.31 ± 0.03	0.33 ± 0.02	0.35 ± 0.06
T15SJ	0.81 ± 0.01a	0.81 ± 0.01a	0.57 ± 0.32a	0.23 ± 0.04	1.50 ± 0.05	0.67 ± 0.31	1.21 ± 0.05	0.29 ± 0.02	0.35 ± 0.01
T25CK	0.18 ± 0.08b	0.18 ± 0.08b	0.07 ± 0.60b	0.43 ± 0.28	4.00 ± 3.35	0.84 ± 0.69	0.96 ± 0.89	3.05 ± 2.46	0.27 ± 0.22
T25SJ	0.79 ± 0.01a	0.79 ± 0.01a	0.52 ± 0.42a	0.14 ± 0.04	1.65 ± 0.17	0.62 ± 0.63	1.31 ± 0.11	0.34 ± 0.05	0.45 ± 0.06

Lowercase letters indicate a significance level of 0.05, and different letters indicate significant differences (*p* < 0.05). ns, no significant difference among treatments (*p* > 0.05).

An increase in Fo (minimum fluorescence) accompanied by a decrease in Fv/Fm (maximum photochemical efficiency of PSII) indicates damage to the PSII reaction centers. As shown in Table 4, the Fo value of T25CK was significantly higher than that of T25SJ, while the Fv/Fm value of T25CK was significantly lower than that of T25SJ. This indicates that tobacco plants without soil agent treatment under 25 °C were significantly affected by disease stress. Under 15 °C, there was no obvious difference between the treatment group and the control group. An increase in ψ_Eo (electron transfer efficiency of PSII) (Equation 2) but a decrease in Fv/Fm implies partial damage to PSII, while the function of the remaining active centers is compensatorily enhanced. The ψ_Eo value of T25CK was significantly higher than that of T25SJ, and the Fv/Fm value of T25CK was significantly lower than that of T25SJ. This suggests that under 25 °C, tobacco plants without soil agent application exhibited a stress response after PSII damage; under 15 °C, there was no obvious difference between the treatment group and the control group. The δ_Ro value (efficiency of electron acceptors at the terminal of PSI) (Equation 3) is related to the efficiency of converting light energy into chemical energy. A higher δ_Ro value indicates a higher degree of openness of PSII reaction centers, stronger activity of the electron transport chain, and better photosynthetic efficiency. From the analysis results in Table 4, the δ_Ro value of T25SJ was higher than that of T25CK (Note: corrected for possible typo in original text), and the difference between the two treatments reached a significant level. A significant decrease in PI_ABS (Equations 4, 5) (performance index of PSII) indicates damage to the overall performance of the photosynthetic system. An increase in Fo accompanied by a decrease in Fm (maximum fluorescence) may reflect the activation of the photoprotective mechanism in tobacco plants under stress conditions. As shown in Table 4, the PI_ABS values of both T25CK and T15CK decreased significantly. Among them, the difference between T25CK and T25SJ reached a significant level, and the Fo value increased significantly while the Fm value decreased significantly at the same time. The difference in PI_ABS values between T15CK and T15SJ was not significant.

**Table 4:** Analysis of photosynthetic activity of tobacco plants under different treatments.

Treatment	Fo	Fm	Fv/Fo	φ_Po (Fv/Fm)	ψ_Eo	δ_Ro	PI_ABS
T15CK	1256.00 ± 182.39bc	6221.50 ± 551.65ab	4.00 ± 0.26a	0.79 ± 0.01a	0.27 ± 0.02b	0.63 ± 0.09b	0.89 ± 0.06b
T15SJ	945.70 ± 336.33c	4892.30 ± 1791.14b	4.11 ± 0.22a	0.81 ± 0.01a	0.30 ± 0.03b	0.67 ± 0.20b	1.20 ± 0.27ab
T25CK	3416.00 ± 92.13a	4132.30 ± 558.26b	0.18 ± 0.14b	0.16 ± 0.09b	0.61 ± 0.12a	0.66 ± 0.39b	0.03 ± 0.02c
T25SJ	1605.90 ± 76.10b	7978.10 ± 174.51a	4.00 ± 0.12a	0.80 ± 0.01a	0.35 ± 0.02b	0.78 ± 0.30a	1.32 ± 0.19a

Lowercase letters indicate a significance level of 0.05, and different letters indicate significant differences (*p* < 0.05).

**Figure 10:**
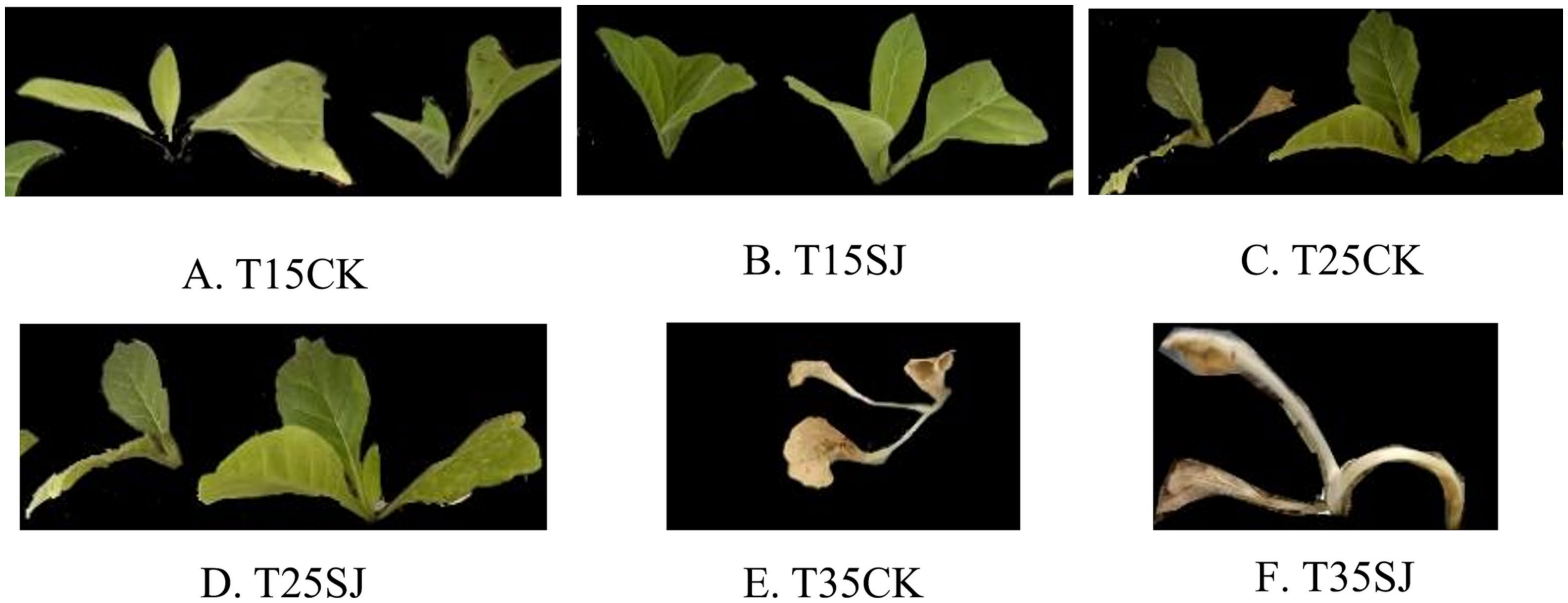
Phenotypic appearance of tobacco plants under different treatments. **(A)** 15 °C without inhibitor application; **(B)** 15 °C with inhibitor application; **(C)** 25 °C without inhibitor application; **(D)** 25 °C with inhibitor application; **(E)** 35 °C without inhibitor application; **(F)** 35 °C with inhibitor application.

Under the temperature condition of 35 °C, whole-plant necrosis occurred in both the T35SJ and T35CK groups, so fluorescence imaging determination was not conducted. At 25 °C, the photosynthetic performance of the treatment without soil agent addition decreased significantly. At 15 °C, there were slight differences in photosynthetic indicators between the treated and control groups, but the differences were not statistically significant (Figure 10).

## 4 Discussion

This study systematically analyzed the effects of a selective soil treatment agent on soil microbial diversity, pathogen inhibition, and tobacco photosynthetic performance under three temperatures (15 °C, 25 °C, and 35 °C). The core innovation lies in verifying a “selective regulation” mechanism that resolves the traditional trade-off between pathogen control and ecological protection. The results confirmed that under different temperature conditions, the application of the soil agent altered the richness of fungal communities, reduced the number of bacterial species while promoting more uniform distribution of remaining bacteria, and exerted a notable inhibitory effect on dominant pathogenic microorganisms. Specifically, at 25 °C, the agent showed significantly better inhibitory effects on two key rhizosphere pathogenic microbial genera, *Pythium* and *Phytophthora*, compared to other treatments. Meanwhile, the photosynthetic system function of tobacco plants characterized by chlorophyll fluorescence parameters such as Fv/Fm (maximum photochemical efficiency of PSII), ΦPSII (actual photochemical efficiency of PSII), and ψ_Eo (electron transfer efficiency of PSII) was significantly improved after the agent application. Taken together, these results together demonstrated the temperature-dependent selective antibacterial effect of the soil agent, as well as the synergistic effect between photosynthetic performance improvement and microbial community regulation.

### 4.1 Mechanism innovation and research value of temperature-dependent antibacterial effect

The agent’s inhibition efficiency against dominant pathogens (e.g., *Fusarium*) was significantly higher at 25 °C (78.6% reduction) than at 15 °C and 35 °C. This result is closely related to the metabolic characteristics of pathogens and the environmental stability of the agent. As reported, the optimal growth temperature of most soil-borne pathogens (e.g., *Fusarium*, *Phytophthora*) is concentrated in the range of 20–30 °C (Arango-Palacio et al., 2024). At this temperature, pathogens exhibit heightened metabolic activity, including enhanced chitin synthesis and zoospore release. While the active components released by the agent, such as atomic oxygen and hydroxyl radicals, can more efficiently target and damage the cell membranes and enzyme systems of pathogens (Velásquez et al., 2018). This is consistent with the result of a marked decline in pathogen abundance observed in the 25 °C treatment group. In contrast, the relative abundance of beneficial bacteria (e.g., *Pseudomonas* and *Bacillus*) increased, indicating they were less affected by the soil agent. This might be related to their robust antioxidant systems or dormant states. Additionally, antibiotic substances secreted by these beneficial bacteria (e.g., 2,4-diacetylphloroglucinol) may further enhance their stress resistance capacity (Mavrodi et al., 2007). ​This combined physiological and metabolic effect thus explains the sustained stability of the bacterial community, as evidenced by a stable Simpson index, in the treated soil groups.

Notably, the agent still exerted a certain inhibitory effect on *Thielaviopsis* (a psychrophilic pathogen) at low temperature (15 °C), while the antibacterial effect weakened at high temperature (35 °C). This may be related to the influence of temperature on the diffusion capacity of the agent and soil adsorption. Low temperature reduced the migration rate of the agent in soil pores, preserving targeting of the cold-adapted pathogens. Reversely, high temperature accelerated the chemical degradation of the agent, resulting in insufficient effective concentration (Kaur et al., 2020; Sangkuhl et al., 2021). This temperature-dependent selective effect revises the traditional view of “disinfectants as non-specific killers”, reveals distinct temperature-responsive differences in the soil agent’s activity against various pathogens while preserving beneficial microbial communities, and provides a theoretical foundation for the targeted prevention and control of complex soil-borne invasive diseases (e.g., the mixed occurrence of root rot and black root rot).

### 4.2 Synergistic effect of photosynthetic performance improvement and microbial community regulation

This study clarified the agent’s cascade effect for the improvement of tobacco growth, which proceeds as follows, firstly selective inhibition of pathogens, then optimization of the microbial community, and subsequently protection of photosynthetic performance. As the results, at 25 °C, the soil agent increased fungal *α*-diversity (Shannon index), enhanced the competitive capacity of beneficial bacteria, and preserved bacterial community stability (stable Simpson index), thus providing a favorable microecological environment characterized by low pathogen abundance and high beneficial microbial enrichment. These results confirmed the viewpoint that “soil health depends on the balance of microbial communities” (Wang et al., 2025; Zhang et al., 2024). The soil agent does not kill microorganisms indiscriminately, but rather creates ecological niches for the proliferation of beneficial bacterial populations by suppressing dominant pathogenic bacteria. Fluorescence imaging data showed that the photosynthetic performance of tobacco plants treated with the agent at 25 °C was significantly better than that of the untreated group. Specifically, the maximum photochemical efficiency (Fv/Fm) and actual photochemical efficiency (ΦPSII) of Photosystem II (PSII) increased, while non-photochemical quenching (NPQ) decreased. This phenomenon may be attributed to the following three mechanisms. The first is the alleviation of root damage. Tobacco plants without agent treatment suffered root structure damage (such as cortical cell necrosis and vascular bundle blockage) due to pathogen infection, which restricted the absorption of water and nutrients, and further led to a decrease in leaf stomatal conductance and a decline in photosynthetic assimilation capacity. This is consistent with previous research results (Wang et al., 2022). The second is the mitigation of oxidative stress. Pathogen infection can induce the outbreak of reactive oxygen species (ROS), while the agent reduces the excessive accumulation of ROS by inhibiting pathogen proliferation, thereby protecting the chloroplast membrane system and PSII reaction centers from oxidative damage (Xiao et al., 2025). The third is the optimization of energy allocation. Untreated tobacco plants need to allocate more energy to disease resistance-related metabolic pathways (such as phenol synthesis), while tobacco plants treated with the agent can prioritize light energy for photochemical reactions, which is manifested by a significant increase in ψ_Eo (electron transfer efficiency) (Zheng et al., 2023). Notably, both the antibacterial effect of the agent and the improvement of photosynthetic performance at 35 °C were weaker than those at 25 °C. This may be attributable to the disorder of plant’s intrinsic metabolism under high-temperature stress (e.g., up-regulation of heat shock proteins and enhanced membrane lipid peroxidation), as well as the accelerated degradation of the soil agent (Du et al., 2021; Lamer et al., 2022). These results suggests that the practical application of the soil agent requires optimizing its application strategies in conjunction with ambient temperature, and thus highlights the necessity of adopting temperature-adapted application regimens.

## 5 Conclusion

This study reveals the temperature-dependent selective regulation mechanism of a soil treatment agent, providing theoretical and practical support for tobacco soil-borne disease management. On one hand, the agent’s inhibitory effect on tobacco black shank and root rot pathogens is significantly temperature-dependent, with 25 °C as the optimal temperature. It selectively targets metabolically active pathogens while preserving beneficial microbes and soil microbial community stability. On the other hand, by inhibiting pathogens and optimizing the rhizosphere microecology, the agent alleviates oxidative stress and root damage, improving tobacco photosynthetic performance, confirming the close link between root health and leaf light energy use efficiency. The “temperature-adapted selective treatment” scheme reduces chemical inputs, resolves the contradiction between “pathogen control” and “ecological protection,” and provides a reference for sustainable crop production. Furthermore, the quantitative relationship model among “soil treatment temperature”, “pathogen abundance”, and “photosynthetic performance” established in this study provides a new perspective for analyzing the interactions among “environment, microorganism, and plant” in soil-borne disease control and supporting crop stress resistance regulation.

## Data Availability

The datasets presented in this study can be found in online repositories. The names of the repository/repositories and accession number(s) can be found at: https://www.ncbi.nlm.nih.gov/bioproject/PRJNA1337005/.

## References

[ref1] An Y. Zhang M. (2024). Advances in understanding the plant-*Ralstonia solanacearum* interactions: unraveling the dynamics, mechanisms, and implications for crop disease resistance. New Crops 1:100014. doi: 10.1016/j.ncrops.2024.100014

[ref2] Arango-Palacio L. Pinzón-Núñez A. M. Hoyos-Carvajal L. Ospina-Galeano D. F. Feria-Gómez D. F. Izquierdo-García L. F. . (2024). Behavior and use of quaternary ammonium-based disinfectants in biosafety protocols against *Fusarium oxysporum* f. sp. cubense race 1 and tropical race 4. Plant Dis. 108, 971–978. doi: 10.1094/pdis-06-23-1138-re, 37877994

[ref3] Aref H. H. (2020). Biology and integrated control of tomato wilt caused by *Fusarium oxysporum lycopersici*: a comprehensive review under the light of recent advancements. Scholars. Direct. 3, 84–99. doi: 10.36959/771/565

[ref4] Bassani I. Larousse M. Tran Q. D. Attard A. Galiana E. (2020). Phytophthora zoospores: from perception of environmental signals to inoculum formation on the host-root surface. Comput. Struct. Biotechnol. J. 18, 3766–3773. doi: 10.1016/j.csbj.2020.10.045, 33304469 PMC7718214

[ref5] Castello I. Baglieri A. Montoneri E. Vitale A. (2025). Utilization of municipal biowaste-derived compounds to reduce soilborne fungal diseases of tomato: a further step toward circular bioeconomy. GCB Bioenergy 17:e70027. doi: 10.1111/gcbb.70027

[ref6] Castello I. D’Emilio A. Danesh Y. Vitale A. (2024). Enhancing the effects of solarization-based approaches to suppress *Verticillium dahliae inocula* affecting tomato in greenhouse. J. Agric. Food Res. 18:101355. doi: 10.1016/j.jafr.2024.101355

[ref7] Chen X. Yuan J. Niu B. Borriss R. Chiang V. L. (2020). Microbial Interactions within Multiple-Strain Biological Control Agents Impact Soil-Borne Plant Disease. Front. Microbiol. 11:585404. doi: 10.3389/fmicb.2020.585404 33162962 PMC7581727

[ref9] del Lorena Rosario C. Pablo B. Fiorela N. Walter G. Erika B. (2023). Exploring the differential impact of salt stress on root colonization adaptation mechanisms in plant growth-promoting rhizobacteria. Plants. 12:4059. doi: 10.3390/plants12234059 38068694 PMC10707856

[ref10] Delitte M. Dubois B. Mahillon J. Debode F. Bragard C. (2024). Monitoring the persistence of *Pseudomonas sivasensis* strain CF10PS3 in cereal fields. Microbiol. Open 13:e005. doi: 10.1002/mbo3.70005, 39552517 PMC11570868

[ref11] Du R. Luo T. Zhang J. Yang L. Li G. (2021). LAMP detection of the blackleg pathogen *Leptosphaeria biglobosa* ‘canadensis’. Crop Prot. 145:105610. doi: 10.1016/j.cropro.2021.105610 33560882

[ref12] Dudung Ahmad S. Dina D. Diah A. (2024). Farmer behaviour of certified high-yielding seed users increases tobacco productivity. Int. J. Multi Discipline Sci. 7:113. doi: 10.26737/ij-mds.v7i2.5203

[ref13] Fry W. (2008). *Phytophthora infestans*: the plant (and R gene) destroyer. Mol. Plant Pathol. 9, 385–402. doi: 10.1111/j.1364-3703.2007.00465.x, 18705878 PMC6640234

[ref14] Gao Z. Yang S. Wang Z. Wang Z. Xi X. He J. . (2022). Effects of different fumigation on continuous cropping soil in peach orchard. Acta Agric. Zhejiangensis 34, 2251–2258.

[ref15] García-Enciso E. L. Benavides-Mendoza A. Flores-López M. Robledo-Olivo A. Juárez-Maldonado A. González-Morales S. (2017). “A molecular vision of the interaction of tomato plants and *Fusarium oxysporum* f. sp. lycopersici,” in *Fusarium* - Plant Diseases, Pathogen Diversity, Genetic Diversity, Resistance and Molecular Markers. (ed.) Askun T.

[ref16] Ge Y. (1996). Epidemiology and control of tobacco black shank. Southwest China J. Agric. Sci. 9, 76–80.

[ref17] Hu T. W. (2009). Tobacco Control Policy Analysis in China: Economics and Health, vol. 82 New York: Social Science Electronic Publishing, 510–511.

[ref18] Kaur R. Das T. K. Banerjee T. Raj R. Sen S. (2020). Impacts of sequential herbicides and residue mulching on weeds and productivity and profitability of vegetable pea in north-western indo-Gangetic Plains. Sci. Hortic. 270:109456. doi: 10.1016/j.scienta.2020.109456

[ref19] Lafferty K. D. (2009). The ecology of climate change and infectious diseases. Ecology 90, 932–933. doi: 10.1890/08-0079.1, 19449681

[ref20] Lamer K. Luke E. P. Mages Z. Leghart E. C. Zhu Z. Puigdomenech Treserras B. . (2022). The impact of heat and inflow wind variations on vertical transport around a supertall building – the one Vanderbilt field experiment. Sci. Total Environ. 851:157834. doi: 10.1016/j.scitotenv.2022.157834, 35944639

[ref21] Larkin R. P. Fravel D. R. (1998). Efficacy of various fungal and bacterial biocontrol organisms for control of *Fusarium* wilt of tomato. Plant Dis. 82, 1022–1028. doi: 10.1094/PDIS.1998.82.9.1022, 30856829

[ref22] Li H. F. Hu Y. Wang C. Liu W. Ma G. Han Q. . (2019). Effects of high temperature and drought stress on the expression of gene encoding enzymes and the activity of key enzymes involved in starch biosynthesis in wheat grains. Front. Plant Sci. 10:1414. doi: 10.3389/fpls.2019.01414 31798603 PMC6863091

[ref23] Li X. Zhang Y. N. Ding C. Xu W. Wang X. (2017). Temporal patterns of cotton *Fusarium* and Verticillium wilt in Jiangsu coastal areas of China. Sci. Rep. 7:12581. doi: 10.1038/s41598-017-12985-1, 28974768 PMC5626778

[ref24] Liu M. Liu X. Song Y. Hu Y. Yang C. Li J. . (2024). Tobacco production under global climate change: combined effects of heat and drought stress and coping strategies. Front. Plant Sci. 15:1489993. doi: 10.3389/fpls.2024.1489993, 39670262 PMC11635999

[ref25] Mavrodi O. V. Mavrodi D. V. Thomashow L. S. Weller D. M. (2007). Quantification of 2,4-diacetylphloroglucinol-producing *Pseudomonas fluorescens* strains in the plant rhizosphere by real-time PCR. Appl. Environ. Microbiol. 73, 5531–5538. doi: 10.1128/aem.00925-07, 17630311 PMC2042083

[ref8] Niu B. Wang W. Yuan Z. Sederoff R. R. Borriss R. (2025). Foliar application of 24-epibrassinolide enhances leaf nicotine content under low temperature conditions during the mature stage of flue-cured tobacco by regulating cold stress tolerance. BMC Plant Biol. 25:77. doi: 10.1186/s12870-025-06080-1 39828684 PMC11744823

[ref26] Panth M. Hassler S. C. Baysal-Gurel F. (2020). Methods for management of soilborne diseases in crop production. Agriculture 10:16. doi: 10.3390/agriculture10010016

[ref27] Papadaki A. M. Bletsos F. A. Eleftherohorinos I. G. Menexes G. Lagopodi A. L. (2017). Effectiveness of seven commercial rootstocks against verticillium wilt and their effects on growth, yield, and fruit quality of tomato. Crop Prot. 102, 25–31. doi: 10.1016/j.cropro.2017.08.006

[ref28] Qurrat Ul Ain F. McComb J. Hardy G. E. S. J. Treena I. B. (2024). Soil amendments for management of Phytophthora root rot in avocado and their impact on the soil microbiome. J. Plant Pathol. 106, 439–455. doi: 10.1007/s42161-024-01604-4

[ref29] Sangkuhl K. Claudio-Campos K. Cavallari L. H. Agundez J. A. G. Whirl-Carrillo M. Duconge J. . (2021). PharmVar GeneFocus: CYP2C9. Clin. Pharmacol. Therap. 110, 662–676. doi: 10.1002/cpt.2333, 34109627 PMC8607432

[ref30] Sbeiti A. A. L. Mazurier M. Ben C. Rickauer M. Gentzbittel L. (2023). Temperature increase modifies susceptibility to Verticillium wilt in *Medicago* spp and may contribute to the emergence of more aggressive pathogenic strains. Front. Plant Sci. 14:1109154. doi: 10.3389/fpls.2023.1109154 36866360 PMC9972977

[ref31] Strasser R. J. Srivastava A. Tsimilli-Michael M. (2000). “The Fluorescence Transient as a tool to Characterize and Screen Photosynthetic Samples,” in Probing Photosynthesis: Mechanisms, Regulation & Adaptation, (eds.) Yunus M. Pathre U. Mohanty P..

[ref32] Velásquez A. C. Castroverde C. D. M. He S. Y. (2018). Plant-pathogen warfare under changing climate conditions. Curr. Biol. 28, R619–R634. doi: 10.1016/j.cub.2018.03.054, 29787730 PMC5967643

[ref33] Wang X. Sun M. Tian L. Yang M. Gao Q. Wang L. I. . (2025). Microbial fertilizers modulate tobacco growth and development through reshaping soil microbiome and metabolome. Microbiol. Spectrum 13:e0260524. doi: 10.1128/spectrum.02605-24, 40401958 PMC12211045

[ref34] Wang Z. Zhang Y. Bo G. Zhang Y. Chen Y. Shen M. . (2022). *Ralstonia solanacearum* infection disturbed the microbiome structure throughout the whole tobacco crop niche as well as the nitrogen metabolism in soil. Front. Bioeng. Biotechnol. 10:903555. doi: 10.3389/fbioe.2022.903555, 35800334 PMC9253565

[ref35] Xiao K. Yang F. Cui W. Li A. Rollins J. A. Guo J. . (2025). A fungal effector promotes infection via stabilizing a negative regulatory factor of chloroplast immunity. Nat. Commun. 16:6970. doi: 10.1038/s41467-025-62326-4, 40730546 PMC12307619

[ref36] Yang B. Feng C. Jiang H. Chen Y. Ding M. Dai H. . (2025). Effects of long-term continuous cropping on microbial community structure and function in tobacco rhizosphere soil. Front. Microbiol. 16:1496385. doi: 10.3389/fmicb.2025.1496385, 40160271 PMC11949956

[ref37] Zhang M. Y. Li H. Miao P. Wang H. Xu M. Yang J.-x. . (2024). Microbial community composition and their activity against *Phytophthora nicotianae* at different growth stages of tobacco. Egypt. J. Biol. Pest Control 34:63. doi: 10.1186/s41938-024-00831-2

[ref38] Zhang Y. Ma L.-J. (2017) Deciphering Pathogenicity of Fusarium oxysporum From a Phylogenomics Perspective in Advances in Genetics, eds. Townsend J. P. Wang Z., vol. 100 (Elsevier, Amsterdam, Netherlands: Academic Press), 179–209. 10.1016/bs.adgen.2017.09.010 29153400

[ref39] Zheng H. Wen F. Zhang C. Luo R. Wu Z. (2023). Novel 1,3,4-Thiadiazole derivatives: synthesis, antiviral bioassay and regulation the photosynthetic pathway of tobacco against TMV infection. Int. J. Mol. Sci. 24:24. doi: 10.3390/ijms24108881, 37240228 PMC10219444

